# Clinical relevance of kallikrein-related peptidase 9, 10, 11, and 15 mRNA expression in advanced high-grade serous ovarian cancer

**DOI:** 10.1371/journal.pone.0186847

**Published:** 2017-11-02

**Authors:** Xiaocong Geng, Yueyang Liu, Sandra Diersch, Matthias Kotzsch, Sabine Grill, Wilko Weichert, Marion Kiechle, Viktor Magdolen, Julia Dorn

**Affiliations:** 1 Clinical Research Unit, Department of Obstetrics and Gynecology, Technical University of Munich, Munich, Germany; 2 Medizinisches Labor Ostsachsen, Dresden, Germany; 3 Institute of Pathology, Technical University of Munich, Munich, Germany; Universidade de Sao Paulo Instituto de Quimica, BRAZIL

## Abstract

KLK9, 10, 11, and 15 may represent potential cancer biomarkers for evaluating ovarian cancer prognosis. In the present study, we selected a homogeneous cohort including 139 patients of advanced high-grade serous ovarian cancer (FIGO stage III/IV) and assessed the mRNA levels of KLK9, 10, 11, and 15 in tumor tissue by quantitative PCR. No significant associations of KLK9, 10, 11, and 15 mRNA with established clinical parameters (residual tumor mass, ascitic fluid volume) were found. Pronounced correlations between KLK10/KLK11 (r_s_ = 0.647) and between KLK9/KLK15 (r_s_ = 0.716) mRNA, but not between other combinations, indicate coordinate expression of distinct pairs of peptidases. In univariate Cox regression analysis, elevated KLK11 mRNA levels were significantly linked with prolonged overall survival (OS; p = 0.021) and progression-free survival (PFS; p = 0.008). KLK15 mRNA levels showed a trend towards significance in case of OS (p = 0.06); KLK9 and KLK10 mRNA expression levels were not associated with patients’ outcome. In multivariable Cox analysis, KLK11 mRNA expression levels, apart from residual tumor mass, remained an independent predictive marker for OS (p = 0.007) and PFS (p = 0.015). Here, elevated KLK15 mRNA expression levels turned out to be significantly related to prolonged OS (p = 0.025) as well. High KLK11 but not the other KLK mRNA levels can be considered as strong independent favorable prognostic factor in this major ovarian cancer subtype.

## Introduction

Ovarian cancer is a common gynecologic cancer among women and is the most lethal one [1]. Ovarian cancer is characterized by asymptomatic nature, lack of effective screening strategy and early metastatic disease. Approximately 75% of the newly diagnosed ovarian cancer cases present already in advanced stage (FIGOIII/IV) resulting in 5-year survival rates of 17%–36% [2]. Until now, the only prognostic factor to be influenced in advanced disease is the amount of residual tumor mass after standard debulking operation [3]. Therefore, identification and validation of biomarkers for prognosis and individualized treatment in ovarian cancer is urgently needed.

Kallikrein-related peptidases (KLKs) are a subgroup of serine proteases encompassing fifteen homologous members. KLKs are involved in broad physiological and pathological processes, such as skin desquamation, semen liquefaction, immune system regulation, and oncogenesis. Several members of the KLK family have been found to be dysregulated in various types of solid cancer, including ovarian cancer. Therefore, they have been extensively studied to explore their use as biomarkers for diagnosis and prognosis in various cancer types. In addition to their utility as biomarkers, numerous studies highlighted their opportunity to be used as therapeutic targets as well [4].

Physiologically, KLK9 is expressed in only few tissues including esophagus, tonsil, and skin. In ovarian cancer, KLK9 expression levels were found to be significantly higher in patients with early disease stage and to display an inverse correlation with CA125 levels. Moreover, KLK9-positive patients have a substantially longer progression-free and overall survival [5]. In breast cancer, higher KLK9 expression is associated with smaller tumor mass, increased patient overall survival, and longer disease-free survival [5]. Conversely, KLK9 has been reported to be associated with higher grade gliomas and may be associated with poor prognosis in this tumor entity [6].

KLK10 is widely expressed in normal human organs, for example breast, prostate, thyroid, testis, ovary, and gastrointestinal tract [7]. On one hand, KLK10 has been proposed to act as tumor suppressor in various types of human cancer, such as breast, esophageal, prostate, testicular, and tongue cancer [8–11]. However, on the other hand, KLK10 has also been reported to be an unfavorable prognostic indicator when overexpressed in ovarian, gastric, colon, endometrial cancer [12–15]. In case of ovarian cancer, Luo and co-workers [13] revealed that high serum levels of KLK10 were significantly associated with serous epithelial type, late-stage, advanced grade tumors, suboptimal debulking, and no response to chemotherapy.

KLK11 is found at the highest level in the prostate, followed by stomach, trachea, skin, and colon [16]. Based on KLK11 serum levels, it has been proposed that ovarian cancer cases can be distinguished from healthy controls, who display very low KLK11 expression [17]. The tumor biological role of KLK11 is not clear at all, since numerous studies have reported a contradictory prognostic impact of KLK11 even in the same type of cancer. Whereas some studies point to an association of elevated KLK11 levels with an unfavorable prognosis in ovarian [18], gastric [19], and lung [20] cancer, others report of a link between low KLK11 levels with poor outcome, again in ovarian [21, 22], gastric [23], and lung [24, 25] tumors.

KLK15, also called prostinogen, is the most recently cloned member of the human kallikrein gene family. KLK15 is robustly expressed in the thyroid, but also detectable in the prostate, salivary glands, adrenal glands, colon, testis, and kidney [26]. In prostate tumors, KLK15 gene expression is significantly higher in cancerous than in noncancerous tissue. KLK15 has been found as an unfavorable prognostic marker for prostate cancer and is upregulated in more advanced and aggressive tumors [27, 28]. Similar findings were seen in ovarian cancer [29]. Here, KLK15 mRNA levels were found to be significantly higher in cancerous tissues compared with benign tumors and, furthermore, elevated KLK15 expression levels were associated with both reduced progression-free and overall survival. However, elevated KLK15 mRNA expression has also been described as a favorable prognostic marker in breast cancer [30].

Although there are some previous reports on expression and potential clinical relevance of KLK9, 10, 11, and 15 in ovarian cancer, in most studies rather heterogeneous patient cohorts were analyzed, including different histological subtypes such as low and high grade serous, mucinous and endometroid tumors. In the present study, we therefore analyzed the expression of KLK9, 10, 11, 15 mRNA in a very homogeneous cohort of patients with advanced high-grade serous ovarian cancer FIGO stage III/IV, encompassing the largest subgroup of patients afflicted with ovarian cancer. mRNA expression levels were determined by newly developed highly sensitive quantitative real-time PCR assays, and the association of KLK mRNA levels with clinical parameters including progression-free and overall survival time of the patients was analyzed.

## Materials and methods

### Patients

139 patients with advanced high-grade serous ovarian cancer FIGO stage III/IV, treated at the Department of Obstetrics and Gynecology, Klinikum rechts der Isar, (TU Munich, Germany) between 1990 and 2012, were enrolled in this study. The study was approved by the local Ethics Committee (Faculty of Medicine, Technical University Munich, Ismaninger Str. 22, 81675 München, Germany, ethikkommission@mri.tum.de; project 1230/04) and written informed consent was obtained from all patients. Median patients’ age at time of surgery was 64 years (range 33–88 years). All patients initially underwent standard stage-related primary radical debulking surgery. Seventy patients (50.4%) were optimally debulked with complete removal of all macroscopically visible tumor manifestations. Following surgery, all of the patients received adjuvant treatment according to consensus recommendations at that time, including platinum-based chemotherapy. None of the patients received any neoadjuvant therapy before primary surgery. Median time of follow-up was 29 months for overall survival (OS; range 2 to 279 months after primary tumor resection) and 20 months for progression-free survival (PFS; range 3 to 279 months). Clinical and histomorphological parameters documented at the time of surgery included histologic subtype, absence or presence of residual tumor mass (0 mm [no visible or palpable tumor left after surgery] versus > 0 mm [any residual tumor left after surgery]) and ascitic fluid volume (≤ 500 ml versus > 500 ml, estimated preoperatively by vaginal ultrasound). During the follow-up time of 5 years, 75 of the 108 patients (with available data for PFS) had relapsed, and 65 of the 125 patients (with available data for OS) had died.

### Real-time polymerase chain reaction

RNA isolation from cell lines and tumor tissue, reverse transcription and cDNA synthesis, real-time polymerase chain reaction details have been described by Ahmed et al. [31].

The following primers (Metabion, Martinsried, Germany) and hydrolysis probes from the Universal Probe Library (Roche, Penzberg, Germany) were used:

KLK9 (numbers for the location of the primers are according to the NCBI entry NM_012315.1): KLK9-forward (209–229): TCCACCTTACTCGGCTCTTCT and KLK9-reverse (288–306): AAGGCGGACCCACAGATAC (reaction concentration: 400 nM each); KLK9-probe: 5’-FAM-GCTGCCCA (reaction concentration: 200 nM); amplicon size: 98 bp.

KLK10 (NM_002776.4, NM_145888.2, NM_001077500.1): KLK10-forward (397–415, 259–277, 298–316): CAGGTCTCGCTCTTCAACG, KLK10-reverse (485–502, 347–364, 386–403): GAGCCCACAGTGGCTTGT (reaction concentration: 400 nM each), KLK10-probe: 5’-FAM-TCCACTGC (reaction concentration: 200 nM); amplicon size: 106 bp. The assay detects the three major KLK10 mRNA transcript variants 1, 2 and 3, all encoding full length KLK10.

KLK11 (NM_006853.2, NM_144947.1): KLK11-forward (150–167, 232–249): GCTTGCTCTGGCAACAGG and KLK11-reverse (201–220, 283–302): AGTGAGGCTTGCACTCGAAC (reaction concentration: 400 nM each); amplicon size: 71 bp. KLK11-probe: 5’-FAM-GAGACCAG (reaction concentration: 200 nM). The assay detects KLK11 mRNA variants 1 and 2, both encoding the identical, full length KLK11 protein.

KLK15 (NM_017509.2, NM_138563.1, NM_138564.1): KLK15-forward (179–197): TCCCTCATCTCCCCACACT, KLK15-reverse (278–297): GTGGTCCGTAGTTGCTCTGG (reaction concentration: 400 nM each), amplicon size: 119 bp. KLK15-probe: 5’-FAM-CTTCCTGC (reaction concentration: 200 nM). The assay detects the three major KLK15 mRNA transcript variants 2, 3 and 4, all encoding full length KLK15.

HPRT1 (NM_000194): HPRT1-forward (218–241): TGACCTTGATTTATTTTGCATACC, HPRT1-reverse (300–319): CGAGCAAGACGTTCAGTCCT (reaction concentration: 400 nM each), HPRT1-probe: 5’-FAM-GCTGAGGA (reaction concentration: 200 nM); amplicon size: 102 bp.

To account for sample heterogeneity, different extraction/conversion efficiencies, and mastermix variations, the PCR efficiency of the KLK9, 10, 11, 15, and HPRT assays was validated by standard dilution series [32]. The efficiency of KLK9, 11, and 15 amplification, but not of KLK10, approximated that of HPRT. Thus, for KLK9, 11, and 15, relative target gene expression was calculated using 2ΔΔCt, where ΔΔCt = ΔCt_sample_ − ΔCt_calibrator_ and ΔCt = Ct_target_ − Ct_HPRT_ [33]. For KLK10, relative target gene expression was calculated using E_KLK10_ − ΔCt_KLK10_∕E_HPRT_ − ΔCt_HPRT_, where ΔCt_gene_ = CT_gene sample_ − CT_gene calibrator_ [33].

### Statistical analysis

All statistical analyses were performed with the SPSS statistical analysis software (version 20.0; SPSS Inc., Chicago, IL, USA). The association of KLKs mRNA expression levels with clinical characteristics of the patients was evaluated using the Chi-square test. Correlations between continuous variables of tumor biological markers were calculated using the Mann-Whitney U test and Spearman rank correlation (r_s_). Associations of tumor biological factors and clinical parameters with patients' survival were analyzed by Cox univariate and multivariable proportional hazards regression models and expressed as hazard ratio (HR) and its 95% confidence interval (95% CI). For the statistical analysis, the observation period was restricted to 60 months. The multivariable Cox regression model was adjusted for established clinical parameters in ovarian cancer such as age, presence of residual tumor mass, and ascites fluid volume. For survival analyses, overall survival (OS) and progression-free survival (PFS) of ovarian cancer patients were used as follow-up end points. Survival curves were plotted according to Kaplan-Meier, using the log-rank model to test for differences. p values ≤ 0.05 were considered statistically significant.

## Results

### KLK9, 10, 11, and 15 mRNA expression in advanced high-grade serous ovarian cancer tumor tissue and their relation to patients’ tumor characteristics

**Fig 1:**
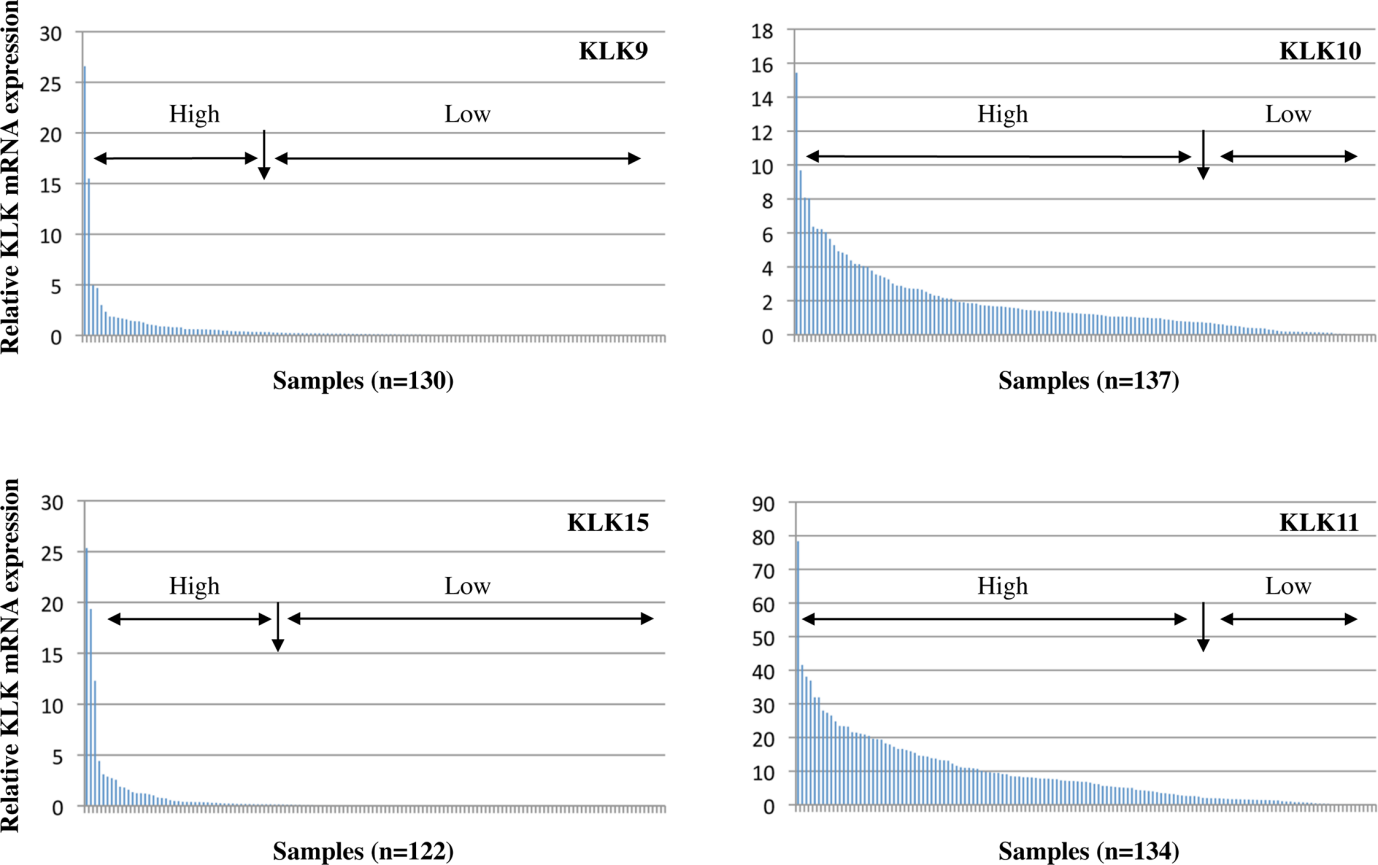
mRNA expression levels of KLK9, 10, 11 and 15 mRNA in advanced high-grade serous ovarian cancer (FIGO III/IV). Cumulative histograms showing relative KLK mRNA expression levels (normalized to HPRT mRNA levels). Most KLK9 and KLK15 samples display very low expression, whereas robust expression is seen for most samples when analyzing KLK10 and KLK11. For further analysis the levels were, thus, dichotomized into low and high expression groups by the 75^th^ percentile for KLK9/KLK15: quartiles 1+2+3 (Q1+2+3) *versus* quartile 4 (Q4) and by the 25^th^ percentile for KLK10/KLK11: Q1 *versus* Q2+3+4.

**Fig 2:**
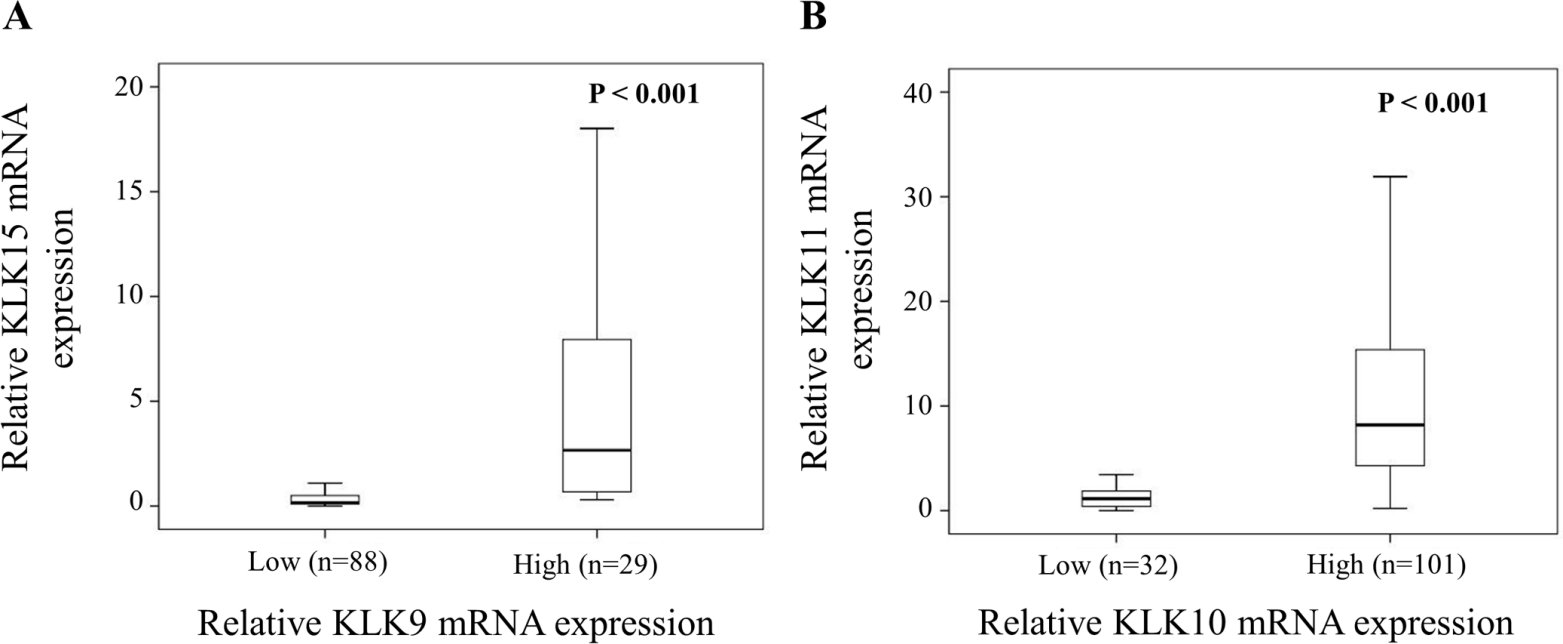
Correlations of KLK mRNA expression levels in tumor tissues of advanced high-grade serous ovarian cancer patients (FIGO III/IV). (A) KLK9 mRNA expression is significantly correlated with KLK15 mRNA levels in tumor tissue. KLK9 mRNA levels were dichotomized into low and high by Q1+2+3 *versus* Q4 and analyzed for association with KLK15 mRNA values (Mann-Whitney test; *p* < 0.001). (B) KLK10 mRNA expression is significantly correlated with KLK11 mRNA levels in tumor tissue. KLK10 mRNA levels were dichotomized into low and high by Q1 *versus* Q2+3+4 and analyzed for association with KLK11 mRNA values (Mann-Whitney test; *p* < 0.001). All KLK mRNA levels were determined by qPCR with normalization to HPRT mRNA levels.

KLK9, 10, 11, and 15 mRNA levels were determined by qPCR in tumor tissues of a homogenous cohort (n = 139) encompassing only patients with advanced serous ovarian cancer FIGO stage III/IV. Most samples display robust expression of KLK10 and KLK11, but only low expression of KLK9 and KLK15 (Fig 1): KLK9 mRNA values ranged from 0.00 to 26.59 (median = 0.14), KLK10 mRNA values from 0.01 to 15.43 (median = 1.24), KLK11 mRNA values from 0.00 to 78.34 (median = 6.97), and KLK15 mRNA values from 0.00 to 25.35 (median = 0.074), respectively. As evident by Spearman correlation analysis, a highly significant positive correlation between KLK9/KLK15 (r_s_ = 0.716, p < 0.001) as well as between KLK10/KLK11 mRNA expression levels (r_s_ = 0.647, p < 0.001), but not between other combinations, was observed. The relationship between low versus high expression levels of these two KLK pairs is also evident in box plot analysis (Mann-Whitney test; p < 0.001; Fig 2). Based on the observed expression pattern of the analyzed KLKs (Fig 1), we categorized the expression levels of KLK9/KLK15 by the 75th percentile in a low-expressing group (encompassing quartiles 1+2+3) versus a high-expressing group (quartile 4), and KLK10/KLK11 by the 25th percentile in a low-expressing group (quartile 1) versus a high-expressing group (quartiles 2+3+4). Table 1 depicts the association between the dichotomized KLK mRNA expression levels in relation to established clinical parameters in ovarian cancer including age, pre-operative ascites fluid volume, and post-operative residual tumor mass. mRNA expression levels of KLKs do not differ significantly in relation to these clinical parameters, except for an association of KLK10 mRNA levels with patients' age (p = 0.014).

**Table 1:** Association between clinical characteristics of advanced ovarian cancer patients (FIGO III/IV) and tumor biological factors.

Clinical parameters	KLK9^a^	KLK10^a^	KLK11^a^	KLK15^a^
	Low/high	Low/high	Low/high	Low/high
**Age**	*p* = 0.686	***p* = 0.014**	*p* = 0.257	*p* = 0.619
** ≤ 60 years**	37/15	8/49	11/45	36/15
** > 60 years**	58/20	26/54	22/56	53/18
**Residual tumor mass**	*p* = 0.165	*p* = 0.457	*p* = 0.763	*p* = 0.847
** 0 mm**	50/14	19/49	17/48	45/17
** > 0 mm**	43/21	15/52	16/51	43/15
**Ascitic fluid volume**	*p* = 0.316	*p* = 0.852	*p* = 0.281	*p* = 0.662
** ≤ 500 ml**	56/17	20/57	21/52	51/18
** > 500 ml**	35/16	13/40	11/43	33/14

^a^Chi-square test (cut-off point: KLK9 = 75^th^ percentile, KLK10 = 25^th^ percentile, KLK11 = 25^th^ percentile, KLK15 = 75^th^ percentile).

Due to missing values, numbers do not always add up to n = 139.

### Association of KLK9, 10, 11, and 15 mRNA expression with overall (OS) and progression-free (PFS) survival in univariate analysis

**Fig 3:**
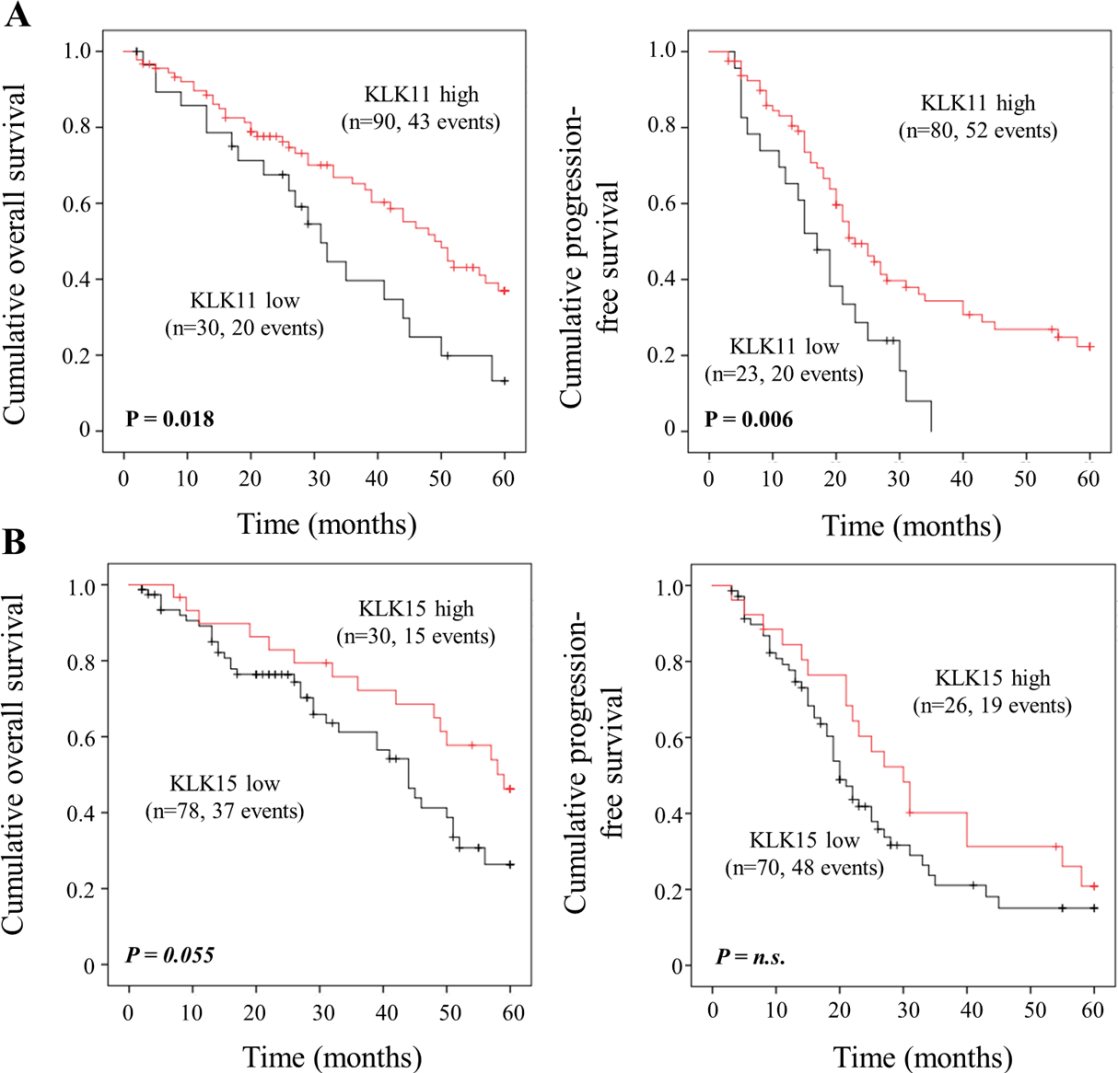
Probability of overall survival (OS) and progression-free survival (PFS) of patients with advanced high-grade serous ovarian cancer (FIGO III/IV) as stratified by KLK11 and KLK15 mRNA expression levels, respectively, in primary tumor tissues. (A) Patients with elevated KLK11 mRNA expression levels show significantly better OS (Kaplan-Meier analysis, *p* = 0.018) (left panel) and progression-free survival (*p* = 0.006) (right panel) than the group of patients with low KLK11 mRNA expression. (B) KLK15 mRNA levels display a trend towards significance (*p* = 0.055) in case of OS (left panel) but not PFS (right panel).

Associations between traditional clinical parameters and KLK mRNA expression levels with patients’ 5-year OS and PFS were analyzed by univariate Cox regression analysis and are summarized in Table 2. Of the clinical factors, residual tumor mass left after debulking surgery and high pre-operative amounts of ascitic fluid indicate a significantly shorter OS and PFS. Elevated KLK11 mRNA levels were found to be a significant predictive factor for both longer OS (HR = 0.53, 95% CI = 0.31–0.91, p = 0.021) and longer PFS (HR = 0.49, 95% CI = 0.29–0.83, p = 0.008), indicating an about two-fold decreased probability of death/progression in the KLK11 high-expressing group. High KLK15 values show a trend towards significance for longer OS (HR = 0.55, 95% CI = 0.30–1.03, p = 0.060). KLK9 and KLK10 mRNA expression levels are neither associated with OS nor with PFS. The findings for KLK11 and KLK15 mRNA expression are visualized by the respective Kaplan-Meier survival curves in Fig 3. Here, high KLK11 mRNA levels are significantly associated with both better OS (p = 0.018) and PFS (p = 0.006), whereas KLK15 values show a trend towards significance in case of OS (p = 0.055).

**Table 2:** Univariate Cox regression analysis of clinical outcome in advanced ovarian cancer patients (FIGO III/IV) with respect to clinical parameters and tumor biological factors.

Clinical parameters	OS			PFS		
	No^a^	HR (95% CI)^b^	*p*	No^a^	HR (95% CI)^b^	*p*
**Age**			0.358			0.627
** ≤ 60 years**	50	1		43	1	
** > 60 years**	75	1.26 (0.77–2.08)		65	1.12 (0.70–1.79)	
**Residual tumor mass**			**<0.001**			**<0.001**
** 0 mm**	64	1		59	1	
** > 0 mm**	59	3.77 (2.18–6.51)		49	2.53 (1.60–4.02)	
**Ascitic fluid volume**			**0.011**			**0.018**
** ≤ 500 ml**	72	1		63	1	
** > 500 ml**	46	1.94 (1.17–3.21)		39	1.78 (1.10–2.87)	
**KLK9 mRNA**^c^			0.311			0.530
** low**	86	1		73	1	
** high**	32	0.75 (0.43–1.31)		28	0.85 (0.5–1.43)	
**KLK10 mRNA**^d^			0.280			0.337
** low**	33	1		27	1	
** high**	92	0.73 (0.41–1.29)		80	0.77 (0.46–1.31)	
**KLK11 mRNA**^d^			**0.021**			**0.008**
** low**	30	1		23	1	
** high**	90	0.53 (0.31–0.91)		80	0.49 (0.29–0.83)	
**KLK15 mRNA**^c^			*0*.*060*			0.147
** low**	78	1		70	1	
** high**	30	0.55 (0.30–1.03)		26	0.67 (0.39–1.15)	

Significant *p*-values (*p* < 0.05) are indicated in bold, trends towards significance (*p* ≤ 0.06) in italics.

^a^ Number of patients

^b^ HR: hazard ratio (CI: confidence interval) of univariate Cox regression analysis

^c^ Dichotomized into low and high levels by the 75^th^ percentile

^d^ Dichotomized into low and high levels by the 25^th^ percentile.

Due to missing values, numbers do not always add up to n = 125 (OS) and n = 108 (PFS).

**Fig 4:**
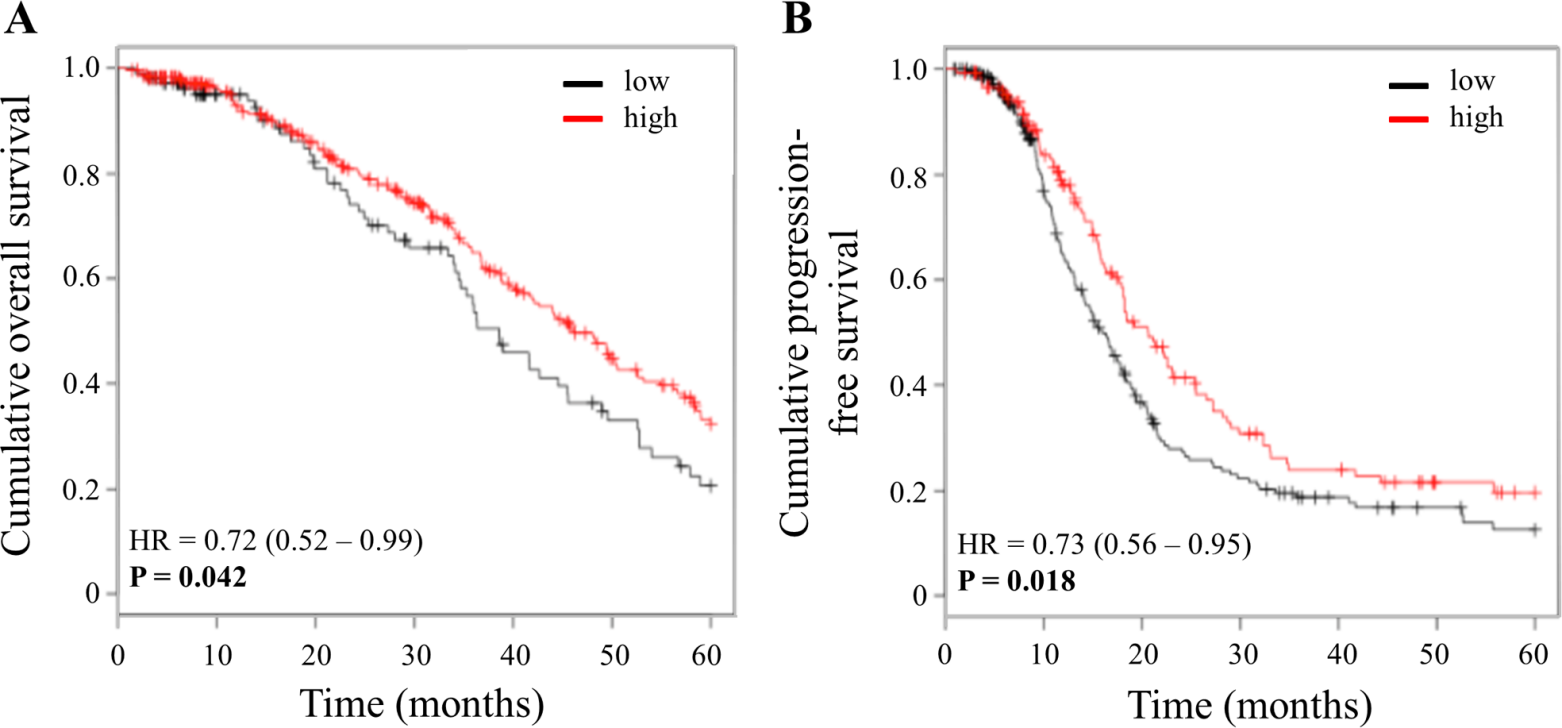
Confirmation of significant associations of KLK11 mRNA expression and patient outcome in an independent, publicly available Affymetrix microarray data set. The microarray data set analyzed for KLK11 mRNA expression (probe ID 205470_s_at) was from The Cancer Genome Atlas (TCGA). Suitable patients were identified by screening the data for the clinical characteristics advanced stage (FIGO III/IV), high-grade (grade 3), serous histological type, platinum-containing chemotherapy, and 5 years’ follow-up. By this, 398 patients were selected in order to assess the association of KLK11 expression with overall survival, and 377 patients with PFS, respectively, applying the assessment tool Kaplan-Meier Plotter [34].

For validation of the results obtained for KLK11 mRNA, we analyzed publicly available Affymetrix-based mRNA data from ovarian cancer patients applying the biomarker assessment tool Kaplan-Meier Plotter [34]. For this in silico analysis, we used the available data set from The Cancer Genome Atlas (TCGA) amounting to 398 patients (OS) and 377 patients (PFS), respectively, selected for the clinical characteristics advanced stage (FIGO III+IV), high-grade (grade 3) serous ovarian cancer patients, who received platinum-based chemotherapy, with 5 years’ follow-up. Kaplan-Meier analysis confirmed that elevated KLK11 mRNA levels are significantly associated with a better prognosis of these advanced, high-grade serous ovarian cancer patients (p = 0.042 (OS); p = 0.018 (PFS); Fig 4).

### Association of KLK9, 10, 11, and 15 mRNA expression with overall (OS) and progression-free (PFS) survival in multivariable analysis

The independent prognostic value of KLK9, 10, 11, and 15 for OS and PFS was studied by multivariable Cox hazard regression analysis, including the factors age, ascites fluid volume, and presence of residual tumor mass (base model) (Table 3). In the base model, residual tumor mass is the only clinical parameter representing a predictive marker for OS (HR = 3.72, 95% CI = 1.84–7.50, *p* < 0.001) and PFS (HR = 2.14, 95% CI = 1.24–3.69, *p* = 0.006), while the pre-operative ascites fluid volume loses its prognostic significance for both OS and PFS when adjusted to multivariable analysis. Among the tumor biological factors (added separately to the base model), KLK11 mRNA levels significantly contribute to the base model for OS (HR = 0.40, 95% CI = 0.20–0.78, *p* = 0.007) and PFS (HR = 0.47, 95% CI = 0.26–0.86, *p* = 0.015). KLK15 mRNA turns out to be independently significant for OS (HR = 0.46, 95% CI = 0.23–0.91, *p* = 0.025). Finally, in the combined model, i.e. KLK11 and KLK15 mRNA values both added to the base model, similar results are obtained: high KLK11 mRNA values are significantly associated with both better OS and PFS, high KLK15 mRNA values remain as an independent factor for OS (Table 3).

**Table 3:** Multivariable Cox regression analysis of clinical outcome in advanced ovarian cancer patients (FIGO III/IV) with respect to clinical parameters and KLK11/KLK15.

Clinical parameters	OS			PFS		
	No^a^	HR (95% CI)^b^	*p*	No^a^	HR (95% CI)^b^	*p*
**Age**			0.225			0.794
** ≤ 60 years**	40	1		36	1	
** > 60 years**	58	1.46 (0.79–2.71)		52	1.07 (0.63–1.83)	
**Residual tumor mass**			**<0.001**			**0.006**
** 0 mm**	53	1		49	1	
** > 0 mm**	45	3.72 (1.84–7.50)		39	2.14 (1.24–3.69)	
**Ascitic fluid volume**			0.798			0.430
** ≤ 500 ml**	60	1		54	1	
** > 500 ml**	38	1.09 (0.55–2.18)		34	1.26 (0.71–2.25)	
**KLK11 mRNA**^c^			**0.007**			**0.015**
** low**	25	1		19	1	
** high**	73	0.40 (0.20–0.78)		69	0.47 (0.26–0.86)	
**KLK15 mRNA**^d^			**0.025**			0.220
** low**	70	1		63	1	
** high**	28	0.46 (0.23–0.91)		25	0.70 (0.40–1.24)	

Biological markers were separately added to the base model of clinical parameters: age, residual tumor mass, and ascitic fluid volume. Significant *p*-values (*p* < 0.05) are indicated in bold

^a^ Number of patients

^b^ HR: hazard ratio (CI: confidence interval) of multivariable Cox regression analysis

^c^ Dichotomized into low and high levels by the 25^th^ percentile

^d^ Dichotomized into low and high levels by the 75^th^ percentile.

## Discussion

In this study, we, for the first time, comprehensively quantified tumor-relevant KLK expression levels in tumors of a homogenous patient cohort suffering of advanced high-grade serous ovarian cancer (FIGO stage III/IV) and assessed the biomarkers’ impact on the course of the disease. In previous studies, KLK9 expression in ovarian cancer has been observed to be significantly higher in patients with earlier stages and low-grade tumors, implicating that KLK9 could be associated with subtypes displaying a less aggressive tumor disease [5]. In the present study, we demonstrate that KLK9 and KLK15 both display low mRNA expression levels in most of the advanced high-grade serous ovarian cancer samples. Moreover, we observed a pronounced coordinate expression of KLK9 and KLK15 on the mRNA level indicating that both proteases may be regulated by a similar mechanism. The genes encoding all of the 15 members of the kallikrein-related serine protease family are located in a single locus spanning approximately 0.3 Mbp on chromosome 19q13.3–13.4, representing the largest contiguous protease cluster in the humane genome. A control by shared enhancer elements, *i*.*e*. by a single locus control region, is unlikely due to the fact that the KLK locus evolved by a series of succeeding gene duplications and displays differential expression of defined, often partially overlapping sets of KLK genes depending on the tissue type. It rather appears that transcription each KLK gene is independently regulated by conserved regulatory elements [35, 36]. Interestingly, expression of the genes within the KLK locus is strongly regulated by androgens, estrogens and other hormones [36]. Co-regulation of KLK gene expression may either require the coordinated binding of hormone receptor/co-transcription factor complexes or may be mediated indirectly via transcription factors, which are hormonally regulated [37]. In fact, in breast cancer cells, KLK9 and KLK15 have both been observed to be up-regulated by androgens [30, 38] and possibly also estrogens [36]. In this tumor type, Yousef et al. [30, 39] found both KLK9 and KLK15 represent independent markers of favorable prognosis in breast cancer.

Concerning the other two analyzed KLKs, prior studies have indicated that KLK10 is elevated in ovarian cancer, with highest levels observed in tumors of more advanced stage [40–42]. KLK11 expression was also significantly higher in ovarian tumor samples than in normal samples [18, 21]. Consistent with these earlier studies, we showed that most of the advanced high-grade serous ovarian cancer samples displayed robust expression of KLK10 and KLK11. Furthermore, our data strongly indicate co-expression of KLK10 and KLK11 on the mRNA level in advanced ovarian cancer, which has already been observed in non-small-cell lung and breast cancer [43, 44]. In breast cancer cells, androgens were shown to synergistically enhance estrogen-dependent expression of KLK10 and KLK11 [45] indicating that coordinate expression of KLK10 and KLK11 may rely on hormone-dependent regulatory mechanisms as well.

Regarding the clinical impact on the course of cancer, KLK9 has been reported to be a prognostic marker in different tumor entities: in glioma patients, stronger KLK9 immunostaining is associated with an unfavorable prognosis [6], whereas in breast cancer, KLK9-positive patients (on mRNA levels) had longer disease-free and overall survival [39]. Also in ovarian cancer, higher KLK9 mRNA levels have been reported to be an independent favorable prognostic marker [5]. In contrast to previous studies, we found that mRNA levels of KLK9 are neither associated with OS nor with PFS in advanced high-grade serous ovarian cancer patients. Similar results were obtained by us applying *in silico* analysis using publicly available Affymetrix-based RNA data [34] from high-grade serous ovarian cancer patients (data not shown). These discrepant findings in ovarian cancer could be due to the different ovarian cancer cohorts analyzed in the studies, as the cohort of the study by Yousef and co-workers [5] included also early stage, low-grade, and other histological types of ovarian cancer patients.

KLK10 protein levels in tumor tissue have been described as an independent, unfavorable prognostic marker not only for late-stage ovarian cancer [41], but also for other types of cancer [12, 14, 15, 41]. Furthermore, Luo et al. [13] determined KLK10 protein levels in sera from a cohort encompassing 146 ovarian cancer patients including patients of all stages, low-grade and high-grade, and different histotypes, and also observed an association of high serum KLK10 protein levels with increased risk for relapse and death in univariate Cox analysis. In addition, via application of KLK10-targeting miRNAs, White et al. [46] suppressed KLK10 expression in OVCAR-3 ovarian cancer cells which resulted in a distinct decrease of cell proliferation in these cells. These results, indicating a tumor-supporting role of KLK10, are in line with the finding that KLK10 represents an unfavorable prognostic biomarker in ovarian cancer [46]. However, in the present study, we did not find any significant association between KLK10 mRNA levels and OS or PFS. Again, *in silico* analysis of publicly available Affymetrix-based mRNA data [34] support our results (data not shown). Possible explanations for these discrepant findings may be that, firstly, we assessed KLK10 mRNA and not protein levels in tumor tissue; and secondly, our cohort only included patients with advanced stage (FIGO stage III/IV), high-grade serous ovarian cancer, while the cohorts analyzed in the two studies by Luo et al. [13, 41] consisted of mixed ovarian cancer subtypes, which of today’s knowledge may not be compared [47].

In contrast to KLK9 and 10, in the present study, high KLK11 mRNA levels were found to be an independent favorable predictor for both OS and PFS in advanced high-grade serous ovarian cancer. This finding was validated by *in silico* analysis of an Affymetrix-based microarray data set [34]. Several other studies previously reported KLK11 as prognostic biomarker for ovarian cancer as well. Borgono et al. [21] reported a favorable prognostic impact of elevated KLK11 protein levels in ovarian cancer tumors tissues. Conversely, Shigemasa et al. [18] reported that KLK11 mRNA overexpression is associated with poor prognosis in patients afflicted with epithelial ovarian cancer. Notably, however, the study on mRNA levels was conducted in a small, very heterogeneous ovarian cancer cohort (n = 64) of which only 26 were of the serous subtype. Furthermore, about half of the tumors were classified as stage I/II.

Also for the reported results on the clinical relevance of KLK15 by Yousef et al. [48], the analyzed cohort was very heterogeneous: only 76 tumors were categorized as serous papillary, whereas the majority (n = 88) corresponded to other histological subtypes and about 30% of patients with grade 1 or 2 tumors. Surprisingly, in spite of the inclusion of early stage tumors, in only 42% of the cases the tumors were completely resected (50% in our cohort encompassing exclusively advanced cancer patients). These major differences concerning the composition of the cohorts may again be the reason why on one hand, high KLK15 levels were identified as an unfavorable prognostic factor for ovarian cancer [48], whereas in the present study elevated KLK15 mRNA levels were associated with a better overall survival of the patients. Interestingly, also in *in silico* analysis–using the same Affymetrix-based ovarian cancer data set from The Cancer Genome Atlas (TCGA) as for KLK9, 10, and 11 –high mRNA levels were associated as a trend (p = 0.068) with a longer OS of ovarian cancer patients. Last but not least, it is of note that elevated KLK15 was suggested as an independent prognostic factor of prolonged OS and PFS in the subgroup of patients with lower grade and hormone receptor negative breast cancer [30].

Explanations for the potential tumor biological role of KLKs and their prognostic value mainly focus on their ability to cleave ECM proteins and to provoke activation/release of signaling molecules such as transforming growth factor (TGF-β), insulin growth factor-1 (IGF-1), epidermal growth factor receptor (EGFR), or the protease-activated receptors PAR1/2 [31]. Furthermore, KLKs by their ability to activate zymogen forms of other proteases, including other members of the KLK family, can form KLK activation cascades as seen in the central nervous system, where such a tissue-specific cascade is formed [49]. Interestingly, KLK11 is involved in many reciprocal cross-activation events, *i*.*e*. KLK11 can activate pro-KLK5, 6, 12, and 14 and—*vice versa*—pro-KLK11 can be activated by the active counterparts of these KLKs. Moreover, KLK5/6, KLK5/12 represent further KLK pairs participating in reciprocal cross-activation [49]. In immunohistochemical studies, we have previously observed that KLK5 as well as KLK6 protein expression by stromal cells within the tumor tissue of ovarian cancer patients significantly contribute to tumorigenicity [50, 51]. In the present study, we quantified mRNA expression of KLK9, 10, 11, and 15 by qPCR. Thus, by this method, we are not able to dissect whether high KLK11/KLK15 tumor or stromal cell-mRNA expression or both is relevant for the better prognosis of the patients.

Notably, expression of all four KLKs, which were analyzed in the present study, has been reported to distinctly change in many defined subpopulations of various types of cancer (depending on the type of cancer and KLK) when compared to their normal tissue counterparts. Dysregulation of expression, on the mRNA and/or protein level, sometimes leads to completely different, i.e. adverse effects concerning progression and outcome of the respective disease. We now know from molecular pathological findings, that ovarian cancer subtypes differ substantially [47]. Therefore, it would, on one hand, be important to identify the tissue-specific substrates of these proteases to understand the tumor-supporting or -suppressing role within a given tumor tissue (sub-)type. On the other hand, the impact of KLK11 mRNA levels as biomarker can be considered only in the context of the subgroup / type of cancer, in which the clinical relevance of this marker has been proven, because in other cancer (sub-)types the situation could be very different.

## Conclusions

In summary, whereas KLK9 and KLK10 mRNA expression does not display any prognostic power in advanced high-grade serous ovarian cancer (FIGO stage III/IV), high KLK15 and, especially, KLK11 mRNA levels are associated with better outcome. These findings should trigger further investigations to reveal the biological effects of these KLKs in ovarian cancer in the future.
